# Rethinking genomic selection under environmental uncertainty: toward learnable and dynamic environmental representations

**DOI:** 10.3389/fpls.2025.1754446

**Published:** 2026-01-20

**Authors:** Jun Yan, Xueyang Wang

**Affiliations:** Frontiers Science Center for Molecular Design Breeding, State Key Laboratory of Maize Bio-Breeding, National Maize Improvement Center, China Agricultural University, Beijing, China

**Keywords:** environmental representation, envirotyping, explainable AI, G×E interaction, genomic selection

## Introduction

The environmental dimension of crop performance has become both the greatest challenge and the greatest opportunity in modern breeding. Increasing climate volatility, soil degradation, and diverse management practices make phenotypes inherently context dependent. Traditional genomic selection (GS) frameworks—built on linear mixed models—excel at capturing additive genetic effects, but when historical datasets are sparse or infrequently updated, they tend to compress environmental variation into categorical factors or a few fixed covariates (Millet et al., 2019). Such fixed-covariate abstraction often fails to capture the complexity, heterogeneity, and temporality of real-world environments, particularly when trial networks are spatially limited or when weather realizations deviate sharply from historical norms.

Importantly, the challenge of representing environmental heterogeneity is not unique to plant breeding. For several decades, spatial ecology has emphasized that ecological patterns and processes are fundamentally scale-dependent, shaped by spatial structure, heterogeneity, and nonlinear interactions across space and time (Levin, 1992). Levin’s seminal articulation of the problem of pattern and scale highlighted that no single spatial or temporal resolution can fully capture environmental complexity, a principle that remains highly relevant for understanding genotype–environment interactions in agricultural systems.

Recent advances in envirotyping, remote sensing, and phenomics have transformed the environment from an external disturbance into a measurable, data-rich component of the phenotype (Xu et al., 2022). In parallel, machine learning (ML) and artificial intelligence (AI) have demonstrated a growing capacity to infer complex, biologically meaningful traits from partial, indirect, or proxy measurements, effectively learning latent structural and functional representations of plant systems (Guo et al., 2025). Such advances underscore the potential of data-driven models to move beyond explicit feature specification toward representation learning, while also enabling the capture of complex, nonlinear genotype–environment (G×E) relationships directly from data (Crossa et al., 2025; Yan and Wang, 2023). Together, these developments imply that the environment should no longer be treated merely as a nuisance to adjust for, but instead as a learnable representation—a structured modality to be modeled, interpreted, and ultimately designed around.

## The constraints of fixed-covariate G×E frameworks

Classical extensions of genomic selection that explicitly model G×E—typically implemented within linear mixed-model frameworks using best linear unbiased prediction (BLUP)—such as reaction-norm formulations and factor-analytic (FA) structures, have long provided the statistical foundation for multi-environment prediction. In their standard form, environments are incorporated as fixed effects and G×E is modeled as a random deviation with predefined covariance structures, often expressed through Kronecker products between genomic and environmental kernels (Cuevas et al., 2025). When trial structures are balanced and environmental gradients are well represented, these frameworks deliver interpretable and regularly updatable estimates; reaction-norm models, for instance, can incorporate daily weather covariates and be re-estimated as new data arrive (Toda et al., 2024).

Despite their historical success, these parametric frameworks are increasingly strained in the era of high-throughput envirotyping, as datasets outgrow the scale, structure, and assumptions of their original designs. A central limitation lies in the *a priori* specification of G×E functional forms—most commonly linear or low-order polynomial—which can fail to capture threshold behaviors, stage-specific stress responses, and genotype-dependent sensitivities observed under complex or fluctuating environments. These structural constraints are further reflected in declining predictive performance when models are transferred across seasons or geographic regions. Such losses in accuracy are only partly attributable to sampling noise and have been increasingly linked to rigid environmental covariate representations, non-stationary climate trajectories, and confounded genotype-by-year effects that are difficult to resolve without extensive multi-year data (Cooper et al., 2021). Moreover, coarse dimensionality-reduction strategies that summarize weather variables over predefined temporal windows can obscure fine-scale temporal dynamics and interactions that are critical for environment-sensitive responses (Garnica and Ojiambo, 2025).

Consequently, the next generation of GS is set to transcend purely parametric interaction modeling and enter an environmental-representation paradigm, one that autonomously extracts informative environmental features without sacrificing the interpretability or variance-partitioning insights inherited from classical mixed-model theory.

## Learning to represent the environment

### Latent and transferable encodings

Latent representation learning refers to a class of machine learning approaches that learn compact, low-dimensional embeddings from high-dimensional data while preserving the structures most relevant for downstream prediction or inference (Kopf and Claassen, 2021). Modern algorithms increasingly integrate latent representation learning to jointly encode genomic, phenotypic, and environmental inputs. Rather than relying on predefined covariates or hand-crafted interaction terms, these approaches learn compact latent variables that summarize high-dimensional inputs while preserving the structure most relevant for prediction. Technically, these representations are often learned through architectures such as variational autoencoder (VAE), which project heterogeneous data streams into a shared latent space. Within this space, environmental variation can be disentangled from genetic structure, enabling models to generalize across populations, traits, and environments by learning transferable environmental embeddings rather than environment-specific regression coefficients (Zhao et al., 2025). This paradigm replaces explicit regression with implicit understanding: rather than fitting genotype-specific slopes to predefined covariates, the model learns an environmental embedding space that reflects hidden regularities in stress patterns, climate variability, and management differences. Such embeddings form the conceptual bridge between predictive modeling and adaptive breeding under changing climates.

### Multi-modal fusion and dual-extraction architectures

Crop performance is rarely determined by a single data modality. Empirical studies in quantitative genetics, functional ecology, and complex systems consistently show that genotype–environment interactions emerge from nonlinear and hierarchical couplings across genetic, physiological, and environmental layers (Crossa et al., 2025; Levin, 1992). Genomic, environmental, and physiological layers interact in hierarchical and nonlinear ways. Multi-modal fusion frameworks therefore use dual-extraction encoders to process each modality independently before integrating them through attention or gating mechanisms (Ren et al., 2024). This approach achieves two goals simultaneously: it preserves the identity and scale of each data type, and it enables the model to learn cross-modal synergies—such as how temperature and soil moisture jointly influence allelic expression or how management practices modify genetic potential. Attention weights or gating coefficients act as interpretable signals, revealing which environmental features and temporal windows most influence trait expression. Thus, these architectures transform opaque black-box predictions into biologically interpretable hypotheses.

### Temporal and dynamic environmental modeling

Environmental effects are inherently dynamic: the timing, duration, and sequence of stress events often determine yield and stability more than their cumulative magnitude, because plants perceive and integrate environmental cues through nonlinear, developmentally gated, and memory-dependent physiological processes rather than linear accumulation. Static environmental averages obscure these critical temporal dynamics. Recent algorithmic advances incorporate temporal embeddings and gated attention layers to model environmental trajectories as structured sequences of signals acting on distinct developmental states, rather than as undifferentiated time series (Yao et al., 2025). In this context, dynamic modeling captures not only when stresses occur, but which environmental variables matter at which developmental stages, and how their effects depend on prior exposure and spatial context. By explicitly accounting for heterogeneous temporal variables—such as short-term extremes, cumulative stresses, and season-long trends—these models can identify windows of sensitivity and genotype-specific adaptive strategies. When coupled with spatially explicit envirotyping, temporal modeling further enables the joint learning of spatiotemporal environmental structure, reframing the environment as a spatially heterogeneous and temporally dynamic field that interacts with genotype across both dimensions.

## Explainability and automation in environment-aware modeling

As models grow in complexity, interpretability becomes both a scientific and operational necessity. Explainable AI (XAI) techniques are now integral to modern GS pipelines, quantifying the relative influence of environmental and genetic features through Shapley values, attention visualization, or saliency mapping (He et al., 2025; Yu et al., 2025). Such tools bridge the gap between prediction and understanding, enabling breeders to trace how particular environmental drivers affect specific genotypes or traits.

Equally transformative is the emergence of automated machine learning (AutoML) pipelines that unify environmental feature engineering, model selection, and hyperparameter optimization (He et al., 2025). These automated systems not only enhance reproducibility and scalability but also democratize access to advanced modeling, enabling breeding programs to implement AI-driven predictions without specialized expertise. By combining automation with explainability, GS is evolving from a research methodology into a practical decision-support system for adaptive breeding.

## From environmental covariates to multi-modal environmental representations

The emerging concept of environmental omics represents a frontier in characterizing agroecosystems, focusing on their biological and biochemical composition rather than traditional physical or climatic descriptors. It integrates soil and rhizosphere microbiomes, as well as management-associated biochemical signatures, providing high-dimensional, functionally informative representations of the external environment (Gioti et al., 2024). When such microbial data are independent of plant phenotypes and sampled *in situ*, they can be treated as external environmental variables (Dwivedi et al., 2025). By capturing these diverse molecular layers, environmental omics enables a mechanistic view of plant–environment interactions, offering unprecedented insights into how environmental states may modulate molecular regulation, stress signaling, and adaptive responses.

Modern multi-modal deep learning frameworks can now integrate these diverse inputs—genomic, phenomic, enviromic, and environmental-omic—treating each as an interconnected modality within a unified representational space. This design enables models to uncover latent couplings between molecular responses and external stimuli, providing mechanistic insights into how environmental conditions modulate gene expression, metabolism, and trait outcomes.

Furthermore, aligning these learned environmental embeddings with mechanistic crop models—for instance, mapping latent representations to parameters governing growth, canopy energy balance, or soil–water dynamics—bridges data-driven adaptability with physiological interpretability. Such hybrid systems combine the generalization strength of machine learning with the causal rigor of process-based modeling, representing a decisive step toward biologically grounded, environment-aware genomic prediction.

## Discussion

Despite remarkable methodological progress, fully realizing environment-aware GS remains a formidable challenge. The bottlenecks now extend beyond computation to data harmonization, model generalization, and biological grounding. A central obstacle is the heterogeneity of environmental data: envirotyping datasets differ widely in spatial scale, temporal resolution, and metadata completeness. Without standardized descriptors and interoperable formats, even advanced models struggle to learn transferable environmental representations.

At the algorithmic level, generalization under novel or extreme climates remains unresolved. Most models are trained on historical datasets that capture only a subset of future climatic variability. When confronted with novel conditions, their predictive accuracy often collapses, underscoring the need for adaptive, transferable frameworks. Transfer learning and meta-learning provide promising directions by enabling models trained in one environment to reuse learned representations in another (Li et al., 2024, 2025), reducing domain dependence and improving robustness under unseen or rapidly changing climatic conditions.

Another major frontier is the heterogeneity of trait architectures. Highly heritable traits respond well to linear models, whereas low-heritability or stress-dependent traits demand more expressive architectures capable of capturing nonlinear G×E dependencies (Drouault et al., 2025; Guo et al., 2025; Millet et al., 2019). No single model can perform optimally across all objectives; one potential way forward is to deploy Task-Adaptive Routing Frameworks (TARFs)—combining specialized sub-models tailored to specific trait–environment contexts through dynamic gating and routing mechanisms (Bai et al., 2024).

Experimental validation remains critical. Model-derived environmental features should be physiologically interpreted and tested in the field, closing the loop between inference and biological understanding. Advancing environment-aware GS thus requires a cohesive system that combines standardized data, adaptive algorithms, and iterative validation—a pathway toward predictive breeding that not only forecasts performance but also reveals principles of adaptation.

**Figure 1:**
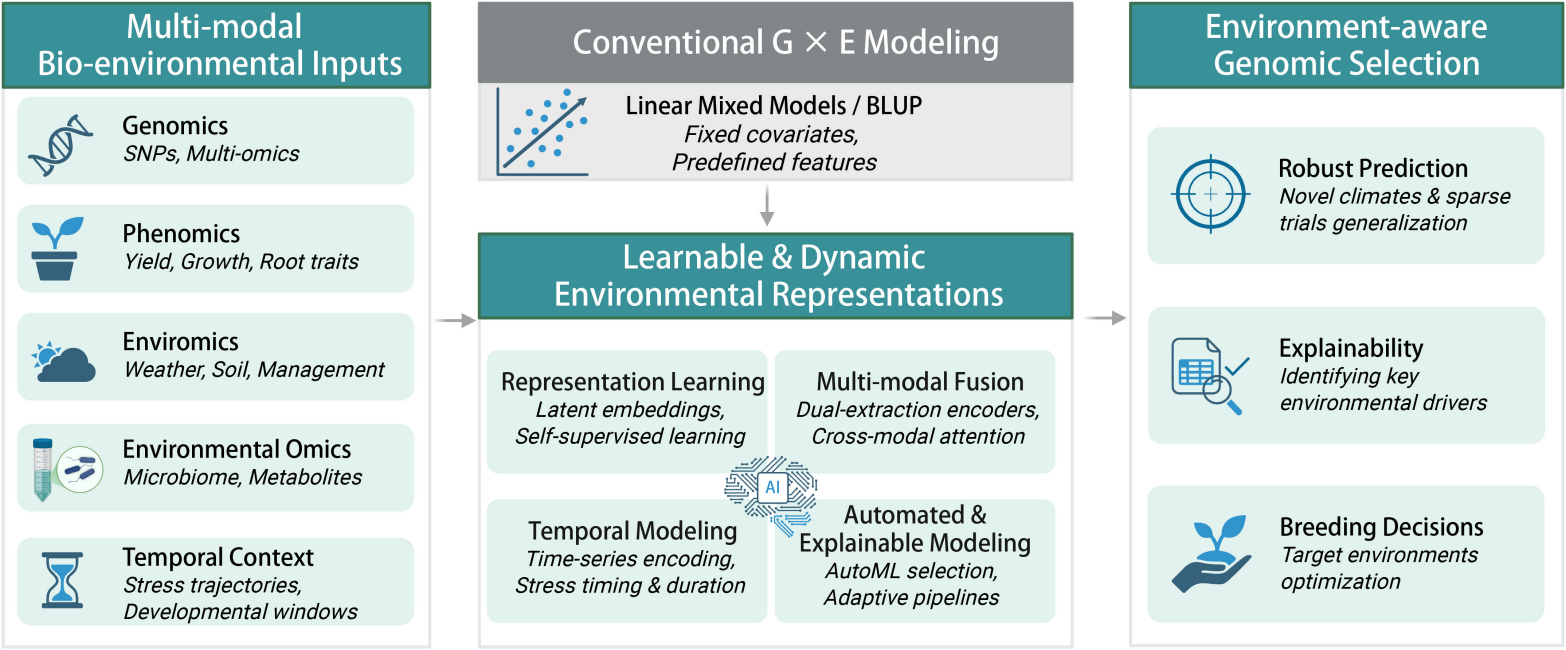
A conceptual framework for environment-aware genomic selection driven by learnable representations.

Building on the conceptual framework illustrated in **Figure 1**, the future of environment-aware GS lies in dynamic, learnable environmental representations rather than static covariates. By integrating multi-modal bio-environmental inputs through deep learning engines, models can perform latent embedding, cross-modal fusion, and temporal modeling, capturing key environmental drivers and sensitive developmental windows. Coupled with automated and explainable AI, this approach enables robust prediction under novel or extreme climatic conditions while enhancing biological interpretability. In this paradigm, predictive breeding moves beyond fitting historical data to uncover principles of adaptation and guide the design of resilient genotypes, truly operationalizing the vision of intelligent, environment-aware selection.
